# Perifosine as a potential novel anti-telomerase therapy

**DOI:** 10.18632/oncotarget.5200

**Published:** 2015-08-15

**Authors:** Brody Holohan, Moriah M. Hagiopian, Tsung-Po Lai, Ejun Huang, Daphne R. Friedman, Woodring E. Wright, Jerry W. Shay

**Affiliations:** ^1^ Department of Cell Biology, UT Southwestern Medical Center, Dallas TX, USA; ^2^ Department of Surgery, UT Southwestern Medical Center, Dallas TX, USA; ^3^ Duke University and Durham VA Medical Center, Durham, NC, USA; ^4^ Center for Excellence in Genomics Medicine Research, King Abdulaziz University, Jeddah, Saudi Arabia

**Keywords:** telomeres, telomerase, targeted therapy, perifosine, CLL

## Abstract

Most tumors circumvent telomere-length imposed replicative limits through expression of telomerase, the reverse transcriptase that maintains telomere length. Substantial evidence that AKT activity is required for telomerase activity exists, indicating that AKT inhibitors may also function as telomerase inhibitors. This possibility has not been investigated in a clinical context despite many clinical trials evaluating AKT inhibitors. We tested if Perifosine, an AKT inhibitor in clinical trials, inhibits telomerase activity and telomere maintenance in tissue culture and orthotopic xenograft models as well as in purified CLL samples from a phase II Perifosine clinical trial. We demonstrate that Perifosine inhibits telomerase activity and induces telomere shortening in a wide variety of cell lines *in vitro*, though there is substantial heterogeneity in long-term responses to Perifosine between cell lines. Perifosine did reduce primary breast cancer orthotopic xenograft tumor size, but did not impact metastatic burden in a statistically significant manner. However, Perifosine reduced telomerase activity in four of six CLL patients evaluated. Two of the patients were treated for four to six months and shortening of the shortest telomeres occurred in both patients' cells. These results indicate that it may be possible to repurpose Perifosine or other AKT pathway inhibitors as a novel approach to targeting telomerase.

## INTRODUCTION

The ends of chromosomes are protected from recognition as double strand DNA breaks by telomeres, nucleoprotein structures on the ends of the chromosomes which consist of telomeric TTAGGG repeats and telomere-binding proteins known collectively as the Shelterin complex (TRF1, TRF2, TIN2, TPP1, POT1 and Rap1) [1]. Because of the end replication problem and difficulty in repairing telomeric DNA damage, telomeres shorten in telomerase-negative human cells at a rate of 50-100 base pairs per cell division [2]. When telomeres become sufficiently short, an unrepairable DNA damage signal initiates a p53/p21-mediated growth arrest [3]. This process is partially prevented in the germ line and certain proliferative stem cell compartments by telomerase, a ribonucleoprotein reverse transcriptase that uses its RNA component, hTR/hTERC, to template the addition of new telomere repeats onto the ends of the telomeres. Because catalytically active telomerase is not expressed in most human somatic tissue, reactivation of telomerase is a barrier that nearly all cancer cells must overcome in order to become a life-threatening tumor [4].

Activation of telomerase is one of the most common perturbations observed in malignancy, present in roughly 90% of tumors [5]. Given this high rate of activation in tumors, low redundancy in telomere maintenance mechanisms and the absence of telomerase activity in most somatic cell compartments, telomerase inhibitors could be effective against a broad spectrum of tumors with minimal effects on most normal tissues. There are a number of telomerase inhibitors in clinical testing, notably Imetelstat, an oligonucleotide competitive inhibitor of the telomerase active site [6]. Recent setbacks in the clinical trajectories of telomerase inhibitors [7] underscore the need for new strategies for telomerase inhibition.

AKT, also known as Protein Kinase B, is a serine/threonine kinase downstream of PI3K, important in a wide variety of signaling events [8]. The protein subunit of telomerase, TERT, has two consensus RXRXXS/T AKT phosphorylation motifs at S227 and S824, both of which are involved in proper nuclear localization of the telomerase holoenzyme [9, 10]. AKT, HSP90, mTOR and S6 kinase form a complex with TERT [11], and Rapamycin has been reported to reduce telomerase activity [12]. These studies provide the rationale that inhibition of AKT may be a viable anti-telomerase strategy.

Perifosine is an orally available alkylphospholipid in phase III clinical trials for multiple myeloma. Perifosine inhibits AKT activity by interfering with the pleckstrin homology domain of AKT, preventing its membrane localization and phosphorylation [13]. Perifosine's oral availability, long half-life in blood (∼100 hours) and low side effect profile [14] make it an excellent candidate for use as an AKT-mediated anti-telomerase therapy, as any anti-telomerase maintenance therapy must be tolerable for long periods of time. Here, we report that low, clinically achievable doses of Perifosine (1.84 μM and 4.6 μM) can induce progressive telomere shortening in a variety of cell lines from multiple tumor cell backgrounds without accelerating telomere shortening in telomerase negative fibroblasts. We also show that long-term Perifosine treatment can reduce soft agar colony formation to a greater extent than acute Perifosine treatment. Further, using both a xenograft model of an anti-telomerase therapy protocol and clinical samples from human CLL patients treated with Perifosine in a phase II clinical trial, we show that long-term Perifosine treatment inhibits telomerase activity and reduced the shortest telomere lengths over time *in vivo*.

## RESULTS

### *In vitro* effects of perifosine on telomerase activity and telomere length

**Figure 1:**
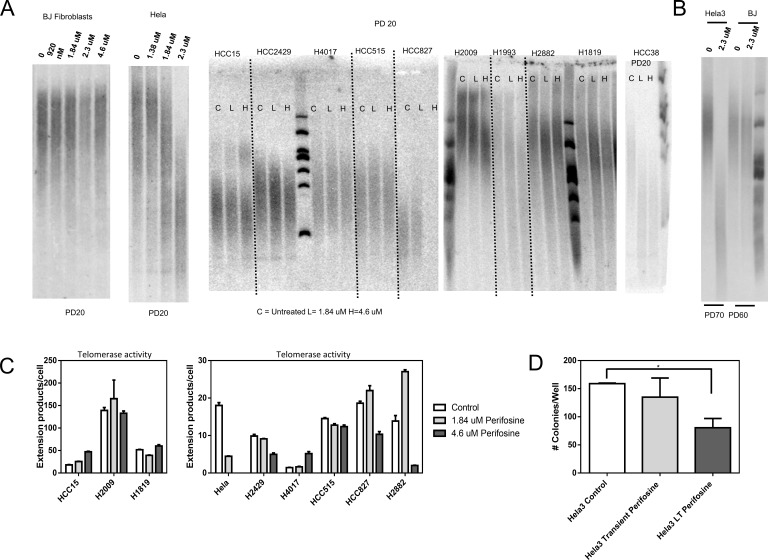
Perifosine induces telomere shortening *in vitro* Initial testing indicated that Perifosine treatment induced telomere shortening over 20 population doublings of exposure in a variety of cancer cell types, while Perifosine did not accelerate telomere shortening in the telomerase negative BJ fibroblast line **A.**, though there was a large degree of heterogeneity in response to Perifosine. Prolonged treatment with Perifosine (70 population doublings) led to further telomere shortening in Hela cells, while not accelerating shortening in BJ cells **B.**, indicating that long-term treatment with Perifosine could result in continuous telomere shortening. Perifosine treatment had variable effects on telomerase enzymatic activity **C.**, measured as number of telomerase extension products per cell input, though changes in enzymatic activity were not always consistent with changes in telomere length. Continuous long-term treatment with Perifosine interfered with colony formation and growth in soft agar **D.**, suggesting that it may be a viable anti-telomerase therapy.

To evaluate the effects of Perifosine on telomere length and telomerase activity, we cultured a panel of cell lines exposed to clinically relevant Perifosine concentrations for extended periods of time. Due to the heterogeneity in signaling dependence in cancer cell lines [15], we decided to test the way telomere biology responded to Perifosine in a large panel of cell lines (Table S1). We assayed cell lines treated with 1.84 uM and 4.6 uM Perifosine (1/8^th^ and 1/2 of the Hela LD_50_, respectively) because the blood concentrations from orally-available doses of Perifosine are reported to be the same order of magnitude as the LD_50_ for a number of cell lines including Hela cells [14, 16]. We observed that 12 of 20 cell lines exhibited telomere shortening after PD 20 (Figure 1A) with a high degree of heterogeneity. Perifosine did not alter the normal rate of telomere shortening in the BJ fibroblast telomerase negative cell line, suggesting telomere shortening was telomerase-dependent. Extended Perifosine exposure continued to drive telomere shortening in Hela cells, but did not accelerate telomere shortening in BJ fibroblasts (Figure 1B). Treatment with Perifosine also altered telomerase enzymatic activity in most but not all cell lines tested (Figure 1C). Some cell lines with reduced telomerase activity did not exhibit telomere shortening. Additionally, AKT inhibitor IV, another AKT inhibitor induced telomere shortening upon continuous administration, though two other compounds that target this pathway did not cause telomere shortening (Figure S5).

To determine if Perifosine exposure and concomitant telomere shortening could impact growth parameters via a subset of critically short telomeres, we performed a soft agar colony formation assay on Hela cells that had not been treated with Perifosine (control cells), Hela cells treated with Perifosine during the soft agar colony formation assay (transient treated cells), and Hela cells treated with Perifosine for 70 population doublings prior to the assay and during the assay (long-term treated cells). Long-term Perifosine treatment significantly (*p* = 0.04) reduced the number of colonies per well, while transient treatment with Perifosine did not induce a statistically significant reduction in colony number (Figure 1D).

### Metastatic xenograft test of perifosine as a telomerase inhibitor

Though other telomerase inhibitors have been tested in xenograft models, measurement of the telomeres from human tumor cells separated from mouse support cells extracted from a xenograft has not been reported [17-20]. To determine if Perifosine can influence the growth and telomere dynamics of tumor cells in a more physiologically relevant setting, we developed a human breast cancer xenograft model that allowed long-term primary and metastatic tumor growth, followed by extraction of human cells and measurement of telomeres with minimal mouse cell contamination (see methods). HCC38 breast cancer cells were selected because they respond strongly to low doses of Perifosine *in vitro* and they exhibit very short baseline telomere length (Figure 1A).

**Figure 2:**
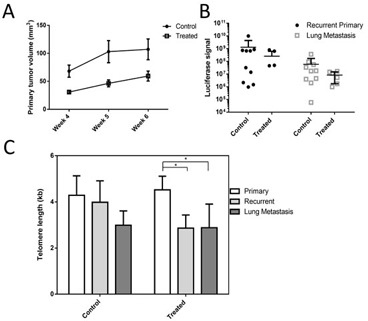
Perifosine reduces primary tumor size and may inhibit telomere maintenance in a xenograft model Perifosine treated primary tumors were smaller on average compared to control tumors **A**. However, neither recurrent primary tumor nor metastatic tumor load were significantly reduced by Perifosine treatment **B.** Telomere length in treated recurrent primary and metastatic tumors was significantly shorter in treated mice **C.**, though there was not a significant difference between control and treated tumors of any type.

Treatment with Perifosine reduced primary tumor size compared with untreated tumors (Figure 2A), though long-term treatment with doses sufficient to induce telomere shortening *in vitro* did not produce a statistically significant reduction in recurrent primary or metastatic tumor burden as measured by luciferase signal intensity (Figure 2B). There was no significant difference of mean telomere length between control and treated tumors of any type, though telomeres from recurrent primary tumors and lung metastatic tumors were significantly shorter compared to primary tumors in the treatment group, indicating that Perifosine may have functioned as a telomerase inhibitor *in vivo* but longer treatments may be required (Figure 2C).

### Telomere length and telomerase activity in CLL tumor samples treated with perifosine

Perifosine is in clinical development in a variety of tumor types such as renal carcinoma and colorectal cancer [21, 22]. Most applications of Perifosine feature cycles of exposure followed by non-exposure, limiting the applicability of these trials as models of Perifosine-mediated telomere shortening. However, one trial [23] tested continuous low-dose Perifosine exposure in CLL patients, and we were able to assay telomere length and telomerase activity in purified tumor samples from a subset of these patients with remaining usable samples.

**Figure 3:**
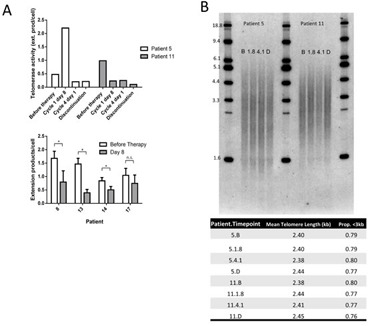
Perifosine reduces telomerase enzymatic activity but does not affect mean telomere length in human CLL Perifosine reduced telomerase enzymatic activity at discontinuation as compared to prior to initiation of therapy in both patients treated for long periods of time, and reduced telomerase activity at day 8 following treatment in four of six patients analyzed **A.** However, Perifosine did not affect mean telomere length measured by TRF in purified tumor cells in either patient treated for extended periods of time **B.** (B = Before therapy, 1.8 = Cycle 1 day 8, 4.1 = Cycle 4 day 1, D = Discontinuation of therapy).

Samples from two patients who had been treated for 4 and 5 cycles (112 and 140 days) were available from before therapy, cycle 1 day 8, cycle 4 day 1 (day 84), and at discontinuation of treatment, while four other patients had samples from before initiation of therapy and at cycle 1, day 8. Telomerase enzymatic activity was reduced at cycle 1 day 8 in four of the six patients tested. Telomerase activity was lower than before initiation of therapy at discontinuation in both patients treated for 4 or more months (Figure 3A). However, mean telomere length in the bulk population did not change with extended Perifosine treatment in these two patient specimens (Figure 3B).

### Universal STELA measurement of the shortest telomeres in Perifosine-treated CLL samples

CLL is composed of varying proportions of proliferative cells depending on a number of poorly-understood factors; for most patients, the majority of the tumor is both non-proliferative and telomerase negative, though periodic reversion to a telomerase positive proliferative state is believed to occur [24]. Due to this slow turnover, it is possible that quantities of time sufficient to produce measurable telomere shortening *in vitro* do not lead to a reduction in mean TRF in spite of the reduced telomerase activity measured. To test if the shortest telomeres in the population were getting shorter, we performed universal Single TElomere Length Analysis (Uni-STELA) in order to quantify the length and dynamics of the shortest telomeres in these samples.

**Figure 4:**
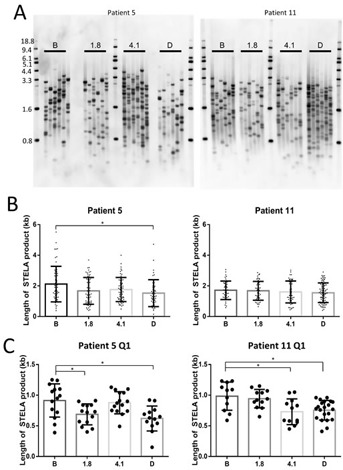
Perifosine induced shortening of the shortest telomeres in continuously treated CLL tumor samples Universal Single Telomere Length Analysis (Uni-STELA) of the shortest telomeres **A.** indicates a reduction in mean STELA product length in one of two patients at discontinuation compared with before therapy **B.**, and a reduction in the length of the shortest quartile (Q1) of STELA products in both patients **C.** (B = Before therapy, 1.8 = Cycle 1 day 8, 4.1 = Cycle 4 day 1, D = Discontinuation of therapy).

There was a significant reduction in total Uni-STELA product length in patient 5, while there was no change in patient 11 (Figure 4A and 4B). However, there was a reduction in the mean length of the shortest quartile of Uni-STELA products in both patients, indicating that the shortest telomeres were indeed becoming shorter (Figure 4C). This is important since it is the shortest telomeres that will result in inhibition of cell growth or induction of apoptosis [25].

## DISCUSSION

This work has shown that Perifosine, an AKT inhibitor, can prevent telomere maintenance *in vitro*, in xenograft models and in CLL patients continuously treated with Perifosine. Perifosine did not increase the normal rate of telomere length reduction in telomerase negative cells, indicating that the reduction in telomere length was telomerase-dependent in cancer cells. HCC4017 did not have telomere shortening in response to Perifosine, and because of its very low baseline activity we evaluated it for the presence of c-circles, a marker for Alternative Lengthening of Telomeres (ALT) [26]. HCC4017 had c-circle abundance roughly equal to SW13, a cell line known to utilize ALT (Figure S6), suggesting that in this case a telomerase-dependent telomere shortening effect of Perifosine may have been prevented because HCC4017 uses ALT to maintain telomeres. Furthermore, Perifosine may impact telomere length through multiple mechanisms due to the heterogeneity in response in cell lines *in vitro* and that a number of cell lines exhibited telomere shortening with continuous Perifosine treatment without a reduction in telomerase activity.

Inhibition of telomerase activity occurred in some lines that exhibited telomere shortening in response to Perifosine, though a change in telomerase activity did not universally predict an alteration in telomere length. Both H2009 and H1819 exhibited telomere shortening without a reduction in telomerase activity, suggesting that Perifosine may impair telomere maintenance in some backgrounds in a telomerase enzymatic activity independent manner, such as by regulating telomerase nuclear localization. AKT may modulate telomerase activity through a number of mechanisms, and Perifosine impacts pathways other than AKT, such as the p38 pathway [27], which may help explain some of the varying telomere biological responses to the drug. It is possible that Perifosine's other activities may be responsible for the observed telomere shortening. We found that sub-lethal concentrations of the AKT inhibitor IV also resulted in telomere shortening over time, though cells treated with AKT inhibitor I and the Dual Pathway Inhibitor (two other small molecules reported to inhibit AKT) did not exhibit telomere shortening in a pilot series of experiments (Figure S5), suggesting that AKT-independent activities of the drugs in question may be responsible for the effects on telomerase.

Somatic telomerase activity in mouse cells coupled with the fact that laboratory mouse telomeres are an order of magnitude larger than human telomeres [28] have complicated pre-clinical evaluation of telomerase inhibitors in the past. In this study, we mostly circumvented the problem of mouse cell contamination of tumor samples through brief tissue culture and the induction of mouse cell death with puromycin treatment since the human tumor cell line was puromycin resistant. Because telomere length predominantly changes during cell division, short periods of time in tissue culture are unlikely to alter telomere length, though small changes as a result of 1-4 population doublings in tissue culture may have occurred. A recent study [17] measured telomere length in xenografts treated with Imetelstat without removing mouse cells, and it is possible that their telomere length data could have been biased by mouse telomere contamination. Indeed, the band present above the 18.8 kilobase ladder band in the non-tumor-bearing mouse lung tissue sample in Figure S2 appears in all xenograft samples in that study, and optimization experiments conducted prior to this work indicate that the presence of 20% by mass mouse contamination of a human DNA sample is sufficient to produce a dramatically biased measurement of telomere length (Figure S3). Improved methods to distinguish between murine and human telomeres in xenograft samples such as that presented here should improve understanding of telomere biology in more physiological contexts in the future.

Though there was a difference between primary, recurrent primary and metastatic tumor telomere length within the treated group in the xenograft model, there was not a significant difference in mean telomere length between control tumors and treated tumors, indicating that Perifosine was not sufficient to induce dramatic telomere shortening in this xenograft model during the relatively short treatment period. It is possible that the mouse microenvironment included some stimulus that antagonized the function of Perifosine, such as estrogen or normoxia, both of which have been associated with increased telomerase activity [29, 30]. It is also possible that the observed small differences in telomere length between tumor sites are the result of founder effects from the parent population, though this seems unlikely because telomere length in the primary tumors was longer than subclones of the parental HCC38+GFP+Luc cell line *in vitro* (Figure S1). It remains possible that cells with short telomeres in the cell population may have dropped out during the treatment *in vivo* resulting in a survivorship advantage to cells with longer telomeres.

This is the first study of a telomerase inhibitor used in the clinic to evaluate changes in telomere length over the course of treatment. Telomere length is a vital biomarker of the efficacy of treatment with a telomerase inhibitor, since the aim of anti-telomerase therapy is to impose a replicative limit on tumors through progressive telomere shortening leading to apoptosis. Changes in mean telomere length were modest, 300-400bp reduction in length of the shortest quartile, in response to Perifosine. It is also possible that clonal evolution within the tumor sample may be partially responsible for the effect observed, and follow-up studies that monitor STELA product length in CLL samples treated with drugs that do not modify telomere length will be required to rule out a clonal evolution event as the cause. The number of samples available from this trial was small, and we were unable to rule out possible confounding effects from factors such as lifestyle changes that can result in changes in telomere length [31, 32]. Treatment beginning earlier in the course of the disease and continuing for longer periods of time would be predicted to cause further reduction in telomere length, which is viable given Perifosine's generally mild toxicity profile at the doses used. Shortening of the shortest telomeres is the most important indicator of a replicative limit [25], and the reduction in length in the shortest quartile of telomeres observed in these patients is encouraging. It is possible that Perifosine provided in a setting of minimal residual disease may prevent relapses in specific subsets of patients by imposing a limit on replication.

The reduction in telomerase enzymatic activity demonstrated in purified tumor samples from Perifosine-treated CLL patients, coupled with the observation of a reduction in the length of the shortest telomeres indicates that Perifosine may be a viable anti-telomerase therapy. Interestingly, mild thrombocytopenia and leukopenia were observed in the clinical trials of Perifosine. These toxicities are the same as those observed with another telomerase inhibitor, Imetelstat [33]. In the genetic disorders of impaired telomere maintenance such as Dyskeratosis Congenita [34] similar clinical presentations have been observed. It is possible that this overlap may be due to “on target” effects of the drugs via dysregulated hematopoietic stem cell maintenance via telomerase inhibition, rather than specific idiosyncrasies of the drugs themselves.

One other study has examined telomerase activity in human patients treated with the telomerase inhibitor, Imetelstat, in PBMCs of pediatric solid tumor patients [33]. They observed a decrease in telomerase activity, albeit with a high degree of heterogeneity. Telomerase enzymatic activity in the tumor or PBMCs is a mechanistically relevant biomarker of the efficacy of anti-telomerase therapy that should be examined in greater detail in future studies of telomerase inhibitors.

Perifosine can inhibit telomerase activity *in vitro* heterogeneously depending on a number of factors yet to be discovered, and this telomerase inhibition can be used to limit cell growth. In summary, long-term treatment with Perifosine induced the shortest quartile of telomeres to shorten in both CLL patients treated in excess of 4 months and inhibited telomerase activity in 4 of 6 patients within 8 days of initial treatment. Overall these results suggest Perifosine may be useful as an anti-telomerase therapy.

## MATERIALS AND METHODS

### Cell culture and *in vitro* drug treatment

All cells were cultured at 37° C in 5% CO2, in Media X (HyClone, Logan UT) with the addition of 10% calf serum (HyClone, Logan, UT). Cells were treated with the indicated concentrations of Perifosine with media changes and fresh Perifosine added every 3 days; cells were passaged 1:32 when they approached 100% confluence.

The other AKT inhibitors, AKT inhibitor I, AKT inhibitor IV, and the PDK1/AKT/Flt Dual Pathway Inhibitor (EMD4 Biosciences), were tested under the same conditions as Perifosine using the concentrations indicated, replaced every three days.

HCC38 cells expressing a Luciferase (HCC38 + Luc) construct were obtained from the Brekken lab (UT Southwestern). HCC38 + Luc cells were infected with a lentiviral vector bearing an empty pGipZ plasmid containing GFP and puromycin resistance genes (HCC38+Luc+GFP).

### Soft agar colony formation assay

The soft agar colony formation was performed as in [35] with a number of modifications. The assay was performed in triplicate in 12-well plates, at a density of 1500 cells per well. In addition to the two agar layers, 0.5mL Media X +10% calf serum was added either with the addition of 1.84uM Perifosine (long-term treated and transiently treated cells) or without the addition of Perifosine (control cells). Cells were allowed to grow under these conditions for four weeks, replacing the media with fresh media every three days. At the conclusion of week 4, colonies were imaged and counted with the ImageJ analysis software.

### Xenograft design

The second mammary fat pads of 30 (15 control and 15 treated) 6-8 week old female NOD/SCID IL2G mice (obtained from an on-campus supplier) were injected with 3*10^6 HCC38 breast cancer cells, and tumors were allowed to grow for 2 weeks before Perifosine was administered to the treated group. Primary tumors were measured once weekly in weeks 4 to 6 by digital calipers. Primary tumors were removed after measurement in week 6. Bioluminescent measurements of recurrent primary tumor burden were obtained in week 12. The mice were subsequently sacrificed and lung tumor burden was measured by luciferase activity. Tumor samples were disassociated and cultured for telomere analysis at primary removal in week 6 as well as from both recurrent primary and lung metastatic tumors in week 12. All xenograft experiments were conducted under UT Southwestern IACUC approval.

The treated group was given an initial loading dose of 62.6 mg/kg Perifosine suspended in sterile PBS by oral gavage in the first week of treatment followed by weekly maintenance doses of 35.8 mg/kg Perifosine which are reported to maintain Perifosine blood concentrations between 12 uM immediately following a dose and 5 uM immediately prior to a dose based on previous studies on Perifosine pharmacokinetics in mice [36].

### Explant culture

Following surgical excision, tumor samples were placed into 750 uL of Gibco Minimum Essential Media (MEM; Life Technologies) on ice and transported to the tissue culture facility. Tumor samples were then chopped into small pieces with autoclaved surgical scissors and resuspended in 750 uL MEM containing 0.5 mg/mL Liberase DL research grade (Roche). Tissue was incubated at 37° C for 60 minutes under agitation at 225 RPM. Fragments were further dissociated by repeat pipetting through 10, 5, and 1mL pipettes (5× each), and then filtered through a 200 micrometer nylon mesh. Samples were centrifuged at 1500 × g for 5 minutes, and then resuspended in 10mL Media X + 10% fetal calf serum + 5 ug/mL puromycin and plated into 10cm dishes for cell culture to remove mouse cells. Fresh Media X + 10% fetal calf serum + 5ug/mL puromycin was replaced 3 days following cell culture to remove dead cells. On day 7 of puromycin selection (to remove mouse contaminating cells), culture dishes were washed 2× with 10 mL phosphate buffered saline, and then harvested for TRF analysis by trypsinization (5 minutes).

### Surgical procedure

For orthotopic mammary fat pad xenograft injections, mice were anesthetized with 2% continuous isoflurane and the area of the incision was thoroughly cleaned with 70% ethanol and betadine. A small incision was made over the right axillary fat pad and 3 × 10^6^ HCC+Luc+GFP cells suspended in 50uL autoclaved PBS+EDTA were injected into the fat pad using a 30-gauge needle. The incision was closed with wound clips, and mice were placed in a recovery cage on a heating pad. Mice were given 0.1 mg/kg intraperitoneal buprenorphine analgesia following breathing stabilization postoperatively and 24 hours later. All surgical instruments were autoclaved prior to operating and a hot bead sterilizer was used to sterilize tools between subjects.

The primary tumors were removed using the same anesthesia and pre-surgical conditions as the xenograft injection procedure. A small incision was made in the skin directly overlying the tumor, and the skin/subcutaneous tissue surrounding the tumor was carefully dissected free from the tumor surface circumferentially using both blunt and sharp dissection. The entire macroscopic primary tumor was removed when possible; however in cases where the primary tumor was involving surrounding structures (brachial artery, chest wall or forelimb musculature) a small amount of tumor was left *in situ*. Incisions were closed with wound clips, and mice were placed in a recovery cage on a heating pad, given buprenorphine analgesia, and monitored until ambulatory.

### Bioluminescence imaging

40 mg/mL D Luciferin (Gold Biotech, MO) suspended in autoclaved PBS was injected subcutaneously in mice anesthetized with 2% continuous isoflurane. Mice were placed on a heated imaging stage for ten minutes, and then imaged using the IVIS Lumina imaging system (Perkin Elmer, MA). Luciferase signals were quantified using the manufacturer's software. Mice were sacrificed immediately following acquisition of the recurrent primary tumor image in week 12; lungs were removed and imaged under the same conditions.

### Animal care

Animals were housed in a pathogen-free facility and the animal protocols were approved by the Institutional Animal Care and Use Committee (IACUC) at the University of Texas Southwestern Medical Center.

### Telomere length and telomerase enzymatic activity assays

Terminal restriction fragment assays were performed as in [37]. The droplet-digital TRAP (ddTRAP) assay was performed as described in [38]. The c-circle assay was performed as described in [26]. Universal STELA was performed as in [39], with the exception that a pre-lengthened, pre-annealed form of the panhandle primer pair was used in order to omit the fill-in step. Panhandle oligos were pre-annealed by heating at 95°C for 5 minutes, followed by cooling to 25°C at a rate of 1°C per minute in New England Biolabs buffer 2 (New England Biolabs, MA). The sequence of the 42-mer panhandle primer was 5′-TGTAGCTGAAGACGACAGAAAGGGCGT GGTGCGGACGCGGG-3′, and the 44-mer was 5′-TACCCGCGTCCGCACCACGCCCTTTCTGT CGTCTTCACGCTACA-3′.

Universal STELA data was quantified using the manufacturer's software (GeneSnap) for the Syngene G-Box (Syngene, MD), using the quantitative methods for determining the molecular weight of western blot data. The TRF ladders run on the gel were used to establish areas of known molecular weight, and the molecular weight of each STELA product was established from the ladders as determined by the software. The automated band identification algorithm provided with the software was used to identify peaks, with minimal human curation to remove obvious errors (e.g. two signals for one band, clearly misidentified bands).

### Human samples

CLL samples (10 million cells/pellet) were purified as part of the previous IRB approved clinical study at Duke University Medical Center [23]. Patients in that study were treated with 50 mg twice daily oral Perifosine for up to six months. Unused samples from the human clinical trial protocol were used for these experiments.

### Statistical analysis

Two-sided t-tests performed with Graphpad Prism 7 software were used to compare group means.

### Study approval

The human and animal studies were conducted with approval from the relevant institutional review boards. Human subjects gave written informed consent prior to their inclusion in the phase II trial.

## SUPPLEMENTARY MATERIAL FIGURES AND TABLE


