# Supplementary material for: Perifosine as a potential novel anti-telomerase therapy
Source: Oncotarget. 2015 Aug 15;6(26):21816–26. doi: 10.18632/oncotarget.5200 (PMC4673128; doi:10.18632/oncotarget.5200)
Supplement: Supplementary file 1 [file oncotarget-06-21816-s001.pdf]

# Perifosine as a potential novel anti-telomerase therapy

**Supplementry Material**

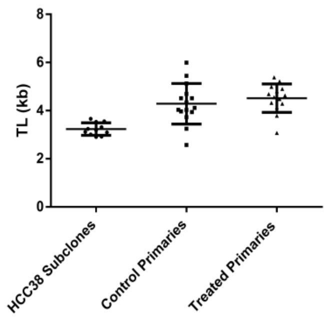


**Figure S1: telomere length in primary tumors compared with subclones of HCC38.** Mean telomere length in both control and treated primary tumors was longer than subclones of the parental HCC38+LUC+GFP population, suggesting that the difference in telomere length between the parent population and the primary tumors was not due to a founder effect.

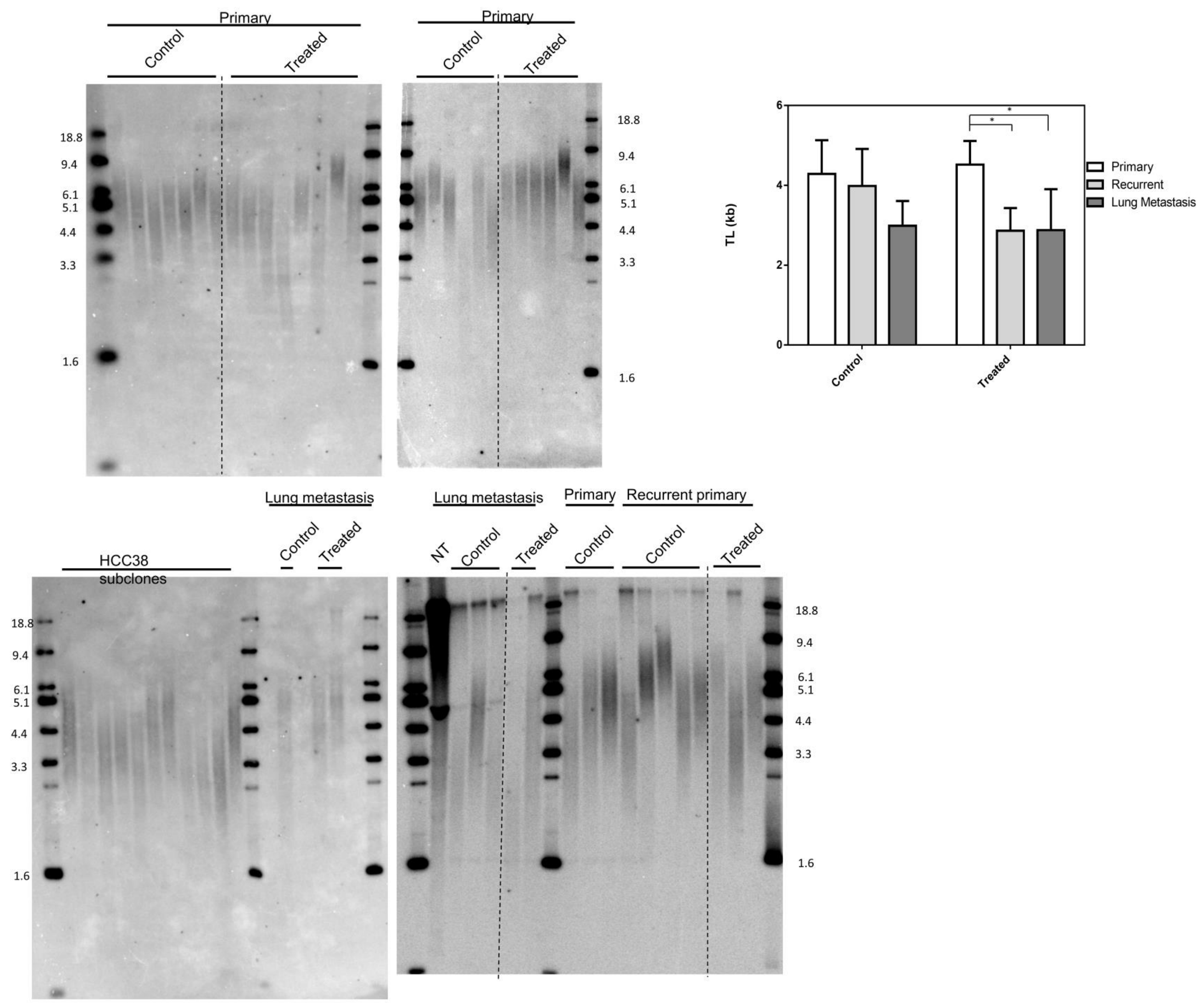


**Figure S2: Terminal Restriction Fragment (TRF) primary data for figure 2.** Mean telomere length was computed as described in [37]; the band indicative of mouse cell contamination is clearly visible in telomeric DNA extracted from a non-tumor-bearing mouse lung (NT, middle right gel).

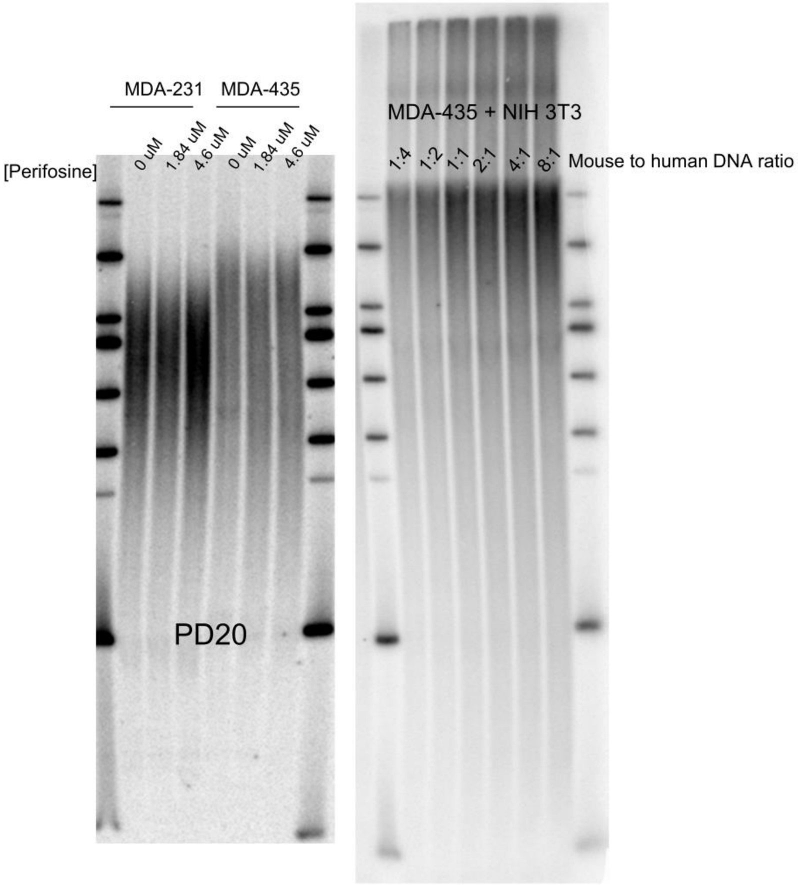


**Figure S3: Small quantities of murine DNA can dramatically bias measurement cancer cell telomere length.** Varying ratios of DNA purified from mouse NIH 3T3 cells to human MDA-MB-435 cells DNA were mixed prior to TRF digestion and analysis. Small quantities of mouse DNA (20% by mass, a ratio of 1:4 mouse to human) were sufficient to bias measurement of MDA-MB-435 cells (right panel) compared with measurement of MDA-MB-435 cells alone (left panel).

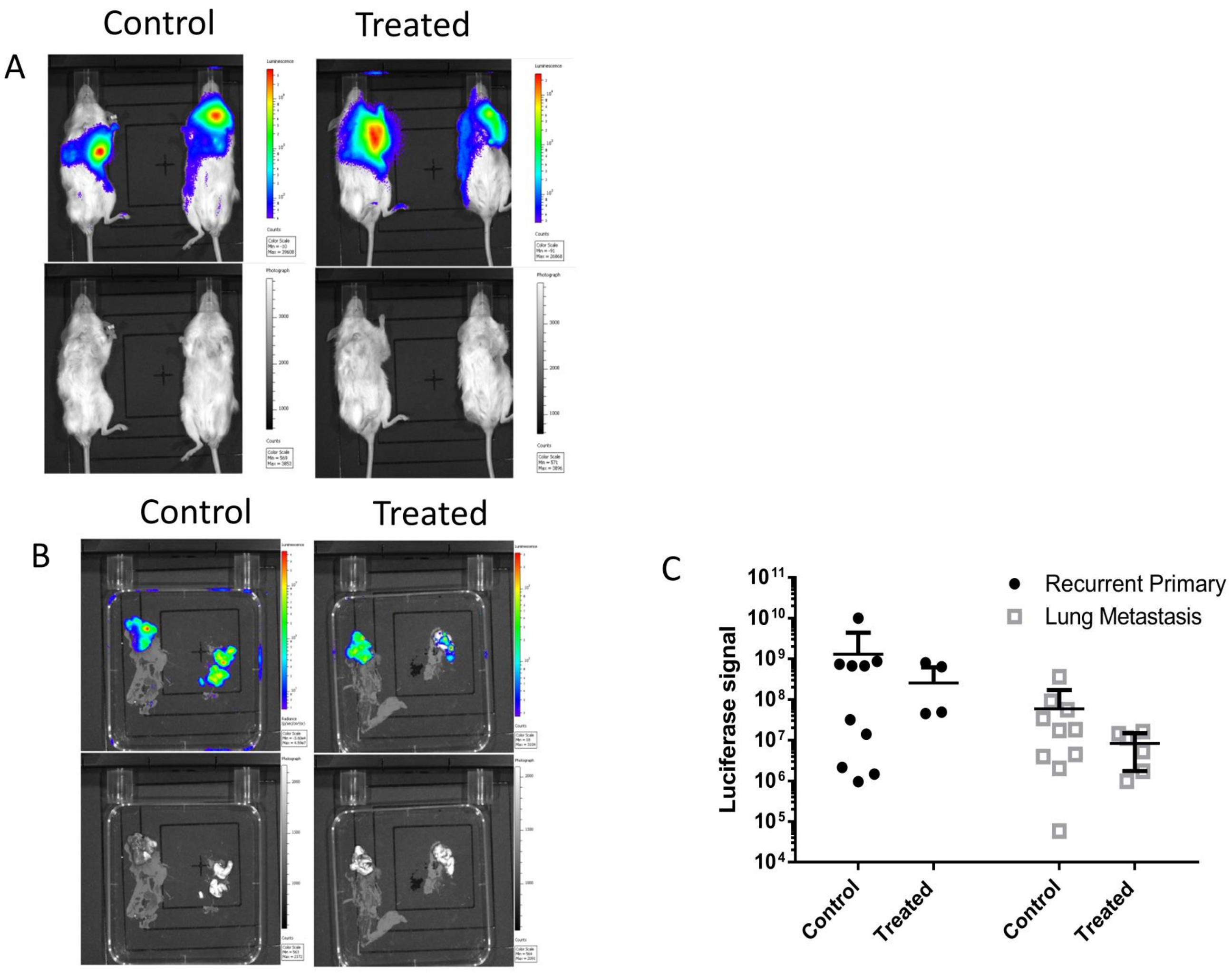


**Figure S4: Representative pictures of bioluminescence signal from recurrent primary and lung metastatic tumors.** There was no statistically significant difference in tumor burden between control and Perifosine-treated mice as measured by bioluminescence signal in either recurrent primary (A) or metastatic tumors (B).

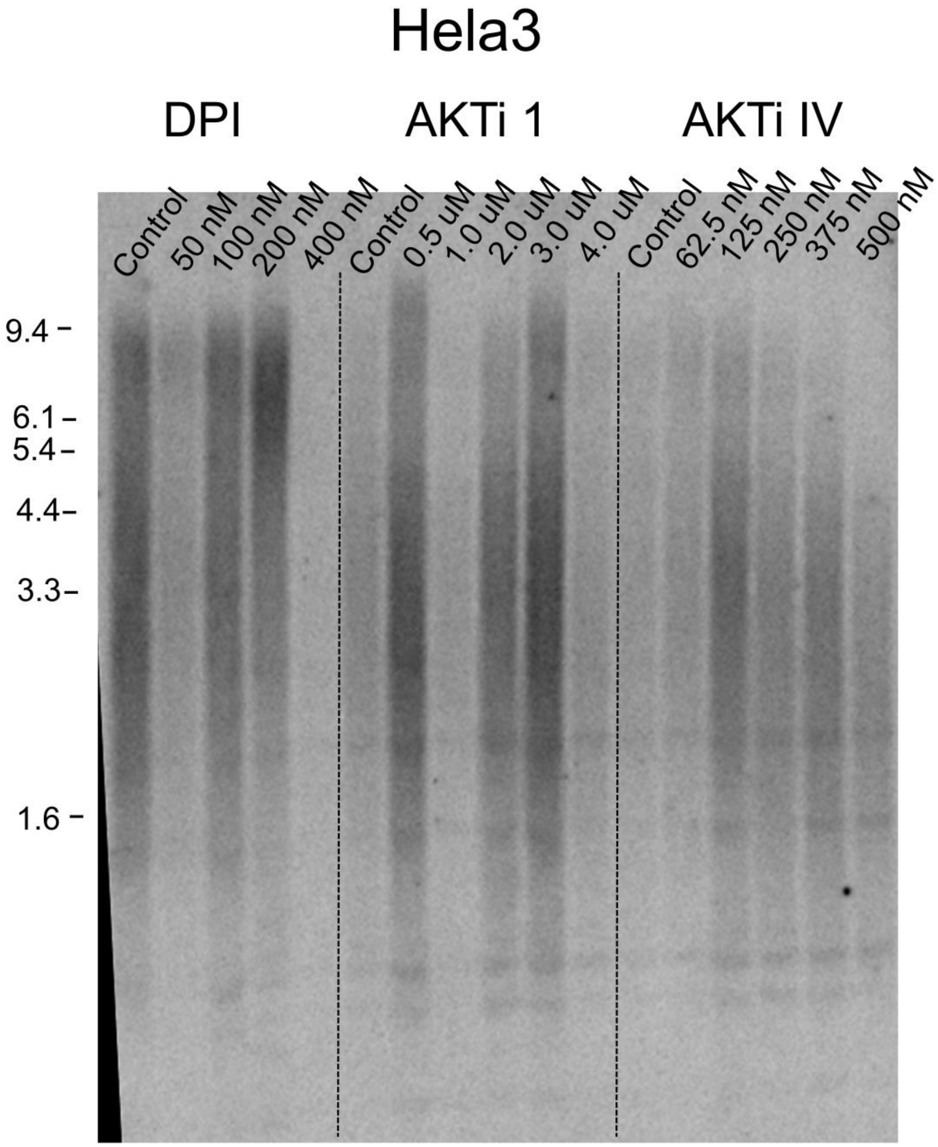


**Figure S5: Tests for telomere shortening in Hela cells exposed to other AKT inhibitors.** Hela cells treated with sub-lethal concentrations of AKT inhibitor IV exhibit telomere shortening over time, while cells treated with AKT inhibitor I and the Dual Pathway Inhibitor did not exhibit telomere shortening.

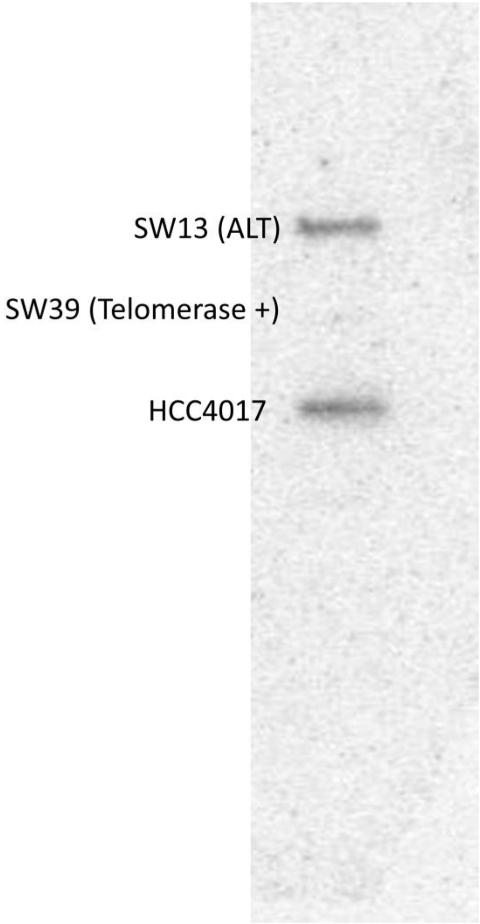


**Figure S6: C-circle assay on HCC4017.** HCC4017 exhibited a number of c-circles consistent with document Alternative Lengthening of Telomeres (ALT)-utilizing cell lines.

**Table S1: Summary of *in vitro* data.** A summary of the cells lines evaluated for changes in telomere length between untreated and the highest measured dose of Perifosine for each cell line (middle) as well as a summary of the change in telomerase enzymatic activity in each cell line (right column).

| Cell Line | Telomere Length | Telomerase activity |
|---|---|---|
| HCC4017 | Unchanged | Increased |
| H2087 | Lengthening | Increased |
| HCC827 | Unchanged | Decreased |
| H2009 | Shortening | Unchanged |
| H2882 | Shortening | Decreased |
| HCC15 | Shortening | Increased |
| H1819 | Shortening | Unchanged |
| HCC515 | Unchanged | Unchanged |
| HCC2429 | Shortening | Decreased |
| Hela | Shortening | Decreased |
| HT1080 | Lengthening | N/A |
| H1993 | Shortening | N/A |
| H2073 | Shortening | N/A |
| HCC95 | Unchanged | N/A |
| MDA-MB-231 | Shortening | N/A |
| MDA-MB-435 | Shortening | N/A |
| DLD1 | Shortening | N/A |
| A375 | Unchanged | N/A |
| HCC38 | Shortening | N/A |
| H1299 | Unchanged | N/A |
